# Retention and Extravasation of Urine in Children

**Published:** 1898-03-19

**Authors:** 


### RETENTION AND EXTRAVASATION OF URINE IN CHILDREN.

In the Lettsomian Lectures delivered before the Medical Society, Mr. John H. Morgan gives the causes of retention of urine in children as being three in number—namely, tumour of the bladder, abscess in the perineum, and the impaction of a stone in the urethra. Again, the two latter conditions are the only causes by which extravasation of urine is produced in young patients, with the exception of rupture of the urethra by falls upon the perineum, and the subsequent obstinate form of stricture which ensues upon that accident. Bearing this in mind the necessity for an examination of the urethra by means of a sound by which the presence of a stone is at once discovered is sufficiently obvious. If (says Mr. Morgan) the stone is arrested in any part of the urethra anterior to the scrotum it can be extracted with suitable forceps, or by means of an incision made directly upon it; but if it be further back it can generally be pushed back into the bladder, and can be removed later or at once by the lithotrite and evacuator. If, however, it remain obstinately in the scrotal or perineal part of the urethra it is easily removed by a median incision. Extravasation must be treated as in adults.

